# Changes of olfactory abilities in relation to age: odor identification in more than 1400 people aged 4 to 80 years

**DOI:** 10.1007/s00405-014-3263-4

**Published:** 2014-09-20

**Authors:** A. Sorokowska, V. A. Schriever, V. Gudziol, C. Hummel, A. Hähner, E. Iannilli, C. Sinding, M. Aziz, H. S. Seo, S. Negoias, T. Hummel

**Affiliations:** 1 Department of Otorhinolaryngology, TU Dresden, Fetscherstrasse 74, 01307 Dresden, Germany; 2 Institute of Psychology, University of Wroclaw, ul. Dawida 1, 50-527 Wroclaw, Poland; 3 Department of Food Science, University of Arkansas, Fayetteville, USA; 4 Department of Otorhinolaryngology, University of Bern, Bern, Switzerland

**Keywords:** Epidemiology, Smell, Sex, Age, Nose, Olfaction, Children

## Abstract

The currently presented large dataset (*n* = 1,422) consists of results that have been assembled over the last 8 years at science fairs using the 16-item odor identification part of the “Sniffin’ Sticks”. In this context, the focus was on olfactory function in children; in addition before testing, we asked participants to rate their olfactory abilities and the patency of the nasal airways. We reinvestigated some simple questions, e.g., differences in olfactory odor identification abilities in relation to age, sex, self-ratings of olfactory function and nasal patency. Three major results evolved: first, consistent with previously published reports, we found that identification scores of the youngest and the oldest participants were lower than the scores obtained by people aged 20–60. Second, we observed an age-related increase in the olfactory abilities of children. Moreover, the self-assessed olfactory abilities were related to actual performance in the smell test, but only in adults, and self-assessed nasal patency was not related to the “Sniffin’ Sticks” identification score.

## Introduction

Olfaction allows us to detect subtle changes in our physical and social environment [1, 2], but sensitivity of this sense varies across individuals [3]. There exist many tests for the assessment of olfactory function [4]; the “Sniffin’ Sticks” test [SST; Burghart Messtechnik, Wedel, Germany; 5–7] is one of the most popular tools.

Sniffin’ Sticks test is a test of nasal chemosensory performance based on a battery of odor-filled felt-tip pens, which are briefly opened to release certain smells. It consists of three tests of olfactory function—odor threshold, odor discrimination, and odor identification; previous works have shown the test–retest reliability of this kit [5, 7]. The normative data of the SST have been established in many countries all over the world [e.g., 6, 8–10] and the SST has already been used in more than 500 studies.

The currently presented large dataset (*n* > 1,400) consists of results that have been assembled over the last 8 years at science fairs using the 16-item odor identification part of the SST. Previous reports on the Sniffin’ Sticks have not been consistent on the existence of sex differences in olfactory sensitivity (e.g., Hummel and collaborators [6] observed sex differences only in some age groups, and Sorokowska and Hummel did not find such differences [10]). Also, although previous reports suggested that olfactory sensitivity of children and older people is lower than sensitivity of young and middle-aged adults [6–10], they have not tested age-related differences in olfactory function of more specific age groups, but rather defined “children” as individuals below 15 years of age and “older people” as people aged above 55. In addition, the seemingly lower olfactory abilities of children have not been fully explored. In this context, the focus was on olfactory function in this age group. Here it is important to note that the current analysis was not meant to produce normative data, but the emphasis was on the reinvestigation of five simple questions that have been addressed in previous studies: (1) Does the ability of odor identification differ by gender? (2) Is there a decrease of olfactory function with age? (3) Do the odor identification scores change in childhood? (4) Is self-assessed olfactory sensitivity related with odor identification ability? and (5) Is self-assessed nasal patency related with odor identification ability?

## Materials and methods

### Participants

In total data were obtained from 1,422 subjects aged 4–80 (356 children aged <16, 203 girls and 153 boys; 696 young adults aged 16–35, 439 women and 257 men; 243 middle-aged adults aged 36–55, 152 women and 91 men; and 127 older adults aged >55, 76 women and 51 men).

Data were collected during various scientific fairs held at the Medical Faculty Campus of the Technical University Dresden (Dresden, Germany). A group of Polish people from Wroclaw (*n* = 28) was also tested to expand the group of the oldest participants (70+). All subjects confirmed that they were in good health. Investigations were performed according to the Declaration of Helsinki and approved by the local ethics committee (EK327082013).

### Procedure

First, the participants were asked to rate their olfactory sensitivity and nasal patency on two 5-point Likert scales ranging from 1 (very good) to 5 (very bad); 3 indicated average. Afterward, trained experimenters assessed olfactory function of participants using the SST 16-item odor identification subtest (Burghart Messtechnik, Wedel, Germany) according to methods published previously [5, 6]. Participants were asked to identify each presented odor from a list of four descriptors; we decided to use only verbal descriptors since previous results have indicated that pictures may not be very helpful in an odor identification task in adults [5]. In children with reading difficulties, the verbal descriptors were read to them by the experimenter. The number of correct answers constituted the identification score. The interval between odor presentations was approximately 20 s. Olfactory function was assessed birhinally.

### Statistical analyses

To explore the olfactory function (defined as the identification score in SST) in relation to age and sex of the subjects, data were submitted to analyses of variance (ANOVA) using the general linear model with main factors: “age group” (defined below) and “sex” (men, women), followed by post hoc Bonferroni *t* tests. We performed three separate analyses on different age groupings:

- To compare our findings to previous reports [5, 6], we separated the subjects into four age groups (A–D): group A: 5–15 years, group B: 16–35 years, group C: 36–55 years, group D: >55 years;
- To further explore the age-related differences in odor sensitivity, we divided our participants on 8 age groups—according to decades;
- We also analyzed the age-related differences in scores of children (aged 15 or less) in detail—their results were analyzed year by year.

Furthermore, subjects were separated into three groups according to (1) their self-assessed olfactory sensitivity and to (2) their nasal patency (I to III): I – subjects who assessed their sense of smell/nasal patency as bad or very bad; II – subjects who assessed their sense of smell/nasal patency as average; and III – subjects who rated their sense of smell/nasal patency as good or very good. The relationships between (1) self-assessed sensitivity and odor identification score, and (2) nasal patency and odor identification score were analyzed using one-way analyses of variance (ANOVA) with factor “self-assessed sensitivity” (in the first analysis) and “nasal patency” (in the second analysis) using Bonferroni post hoc tests. Additionally, the relationship between self-assessed olfactory abilities and self-rated nasal patency was analyzed using Pearson’s *r* correlation.

We undertook two-tailed tests throughout, using STATISTICA ver10 (StatSoft, Inc., Tulsa, USA) with *p* < 0.05 as the level of significance.

## Results

### Effects of sex and age on odor identification

#### Results for 4 age groups (A–D)

Descriptive statistics for groups A, B, C, and D are shown in Table 1.

**Table 1:** Descriptive statistics of the SST identification scores for subjects separated into 4 age groups (*n* number of subjects, *M* mean, *SD* standard deviation, *female* vs. *male* significance of difference between scores of females and males in a given age group)

Age group	Age range (years)	*n*	Score *M*	Score *SD*	Median	10th percentile	90th percentile	Female score *M*	Male score *M*	Female vs. male *p*
A (young children)	<16	356	12.45	2.20	13	9	15	12.64	12.19	0.79
B (young adults)	16–35	696	13.77	1.62	14	12	16	13.83	13.67	1
C (middle-aged adults)	36–55	243	13.79	1.68	14	12	16	13.86	13.66	1
D (older adults)	>55	127	12.12	2.82	12	9	15	12.12	12.12	1

We found significant differences in odor identification abilities between the age groups [significant main effect of “age”: *F* (3, 1,414) = 56.3, *p* < 0.001]. Post hoc Bonferroni *t* tests showed that the groups A and D scored lower than groups B and C (*p* < 0.001). All the remaining differences were not significant (all *p* > 0.05). We did not observe any significant effect of sex for the identification score [*F* (3, 1,414) = 2.72, *p* = 0.10]. Also, we found no significant interaction effect of the factors “sex” and “age group” [*F* (3, 1,414) = 0.61, *p* = 0.61]. To further investigate the sex differences within particular age groups, we conducted four independent samples *t* tests. The differences between men and women were not significant in either of the age groups (see Table 1 for detailed results; all *p*s > 0.05).

#### Results for 8 age groups (1–8)

Descriptive statistics for 8 age groups (decades) can be found in Table 2.

**Table 2:** Descriptive statistics of the SST identification scores for subjects separated into 8 age groups (*n* number of subjects, *M* mean, *min* minimum, *max* maximum, *SD* standard deviation)

Age group	Age range (years)	Total *N*	Score *M*	Score *min*	Score *max*	Score *SD*	Median	10th percentile	90th percentile	Females *N*	Female score *M*	Males *n*	Male score *M*
1	4–9	185	11.88	5	16	2.35	12	9	15	104	12.01	81	11.72
2	10–19	249	13.29	6	16	1.80	14	11	15	157	13.53	92	12.88
3	20–29	525	13.79	5	16	1.53	14	12	16	333	13.88	192	13.63
4	30–39	137	13.71	3	16	2.00	14	12	16	76	13.47	61	14.00
5	40–49	157	13.83	9	16	1.52	14	12	16	99	14.00	58	13.55
6	50–59	71	13.37	6	16	2.20	14	11	16	43	13.42	28	13.29
7	60–69	54	12.48	2	16	2.71	13	10	15	29	12.10	25	12.92
8	70–80	44	11.05	3	16	2.92	11.5	7	15	29	11.48	15	10.20
SUM		1,422								870		552	

We found a significant interaction effect “sex × age” [*F* (7, 1,406) = 2.31; *p* = 0.02], but the effect size was low (*η* ^2^ = 0.01) and post hoc Bonferroni tests revealed no significant sex differences within particular age groups (detailed results of men and women can be found in Table 2).

**Fig. 1:**
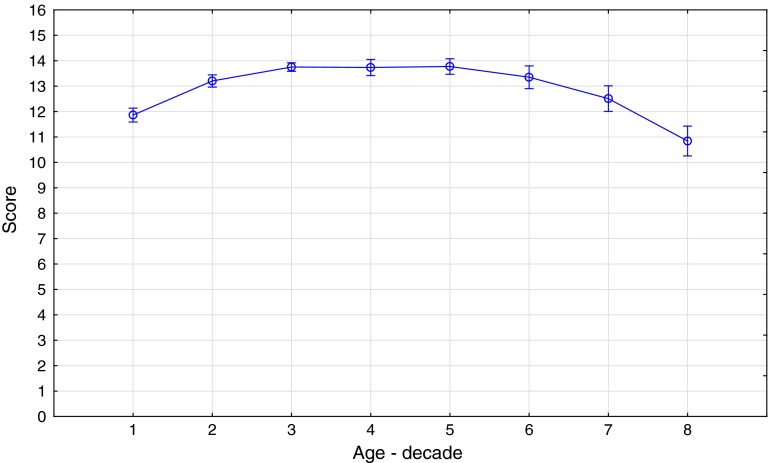
Age-related differences in SST identification scores (means indicated by *circles* and standard deviations indicated by *bars* are shown for age groups 1–8 in decades; For the details of age-decade, please see Table 2)

Comparison of the scores of groups 1–8 revealed significant differences [*F* (7, 1,406) = 32.6, *p* < 0.001] between the age groups. Post hoc Bonferroni tests showed that scores of age group 8 (people aged 70+) were significantly lower than scores of any other group, except for the youngest (<9) children (all *p* < 0.001). Group 1 (children aged 9 and younger) scored significantly lower (*p* < 0.001) than age groups 2–6 (people aged 10–59). Group 7 (people aged 60–69) scored significantly lower (all *p* < 0.001) than age groups 3–5 (people aged 20–49). Additionally, the scores of children from the age group 2 (aged 10–19) were significantly lower (*p* = 0.02) than the scores of people from age group 3 (aged 20–29). All the other differences were not significant (all *p* > 0.05; see Fig. 1). There was no significant main effect of “sex” [*F* (1, 1,406) = 2.45; *p* = 0.12;* η* ^2^ = 0.14].

#### Age-related differences in olfactory function of children

Descriptive statistics of children’s scores are presented in Table 3.

**Table 3:** Descriptive statistics of SST identification test results in children (*n* number of subjects, *M* mean, *SD* standard deviation)

Age (years)	*n*	Score *M*	Score *SD*
4	11	11.45	0.92
5	10	10.70	0.94
6	22	10.73	0.48
7	33	11.67	0.38
8	49	12.22	0.31
9	60	12.42	0.29
10	39	12.36	0.31
11	36	13.64	0.26
12	40	13.08	0.29
13	25	13.20	0.29
14	18	12.94	0.56
15	13	13.38	0.47

We found a significant interaction effect “sex × age” [*F* (11, 332) = 2.00; *p* = 0.03], but the effect size was extremely low (*η* ^2^ = 0.06) and post hoc Bonferroni tests revealed no significant differences between boys and girls of any ages. Comparison of the scores obtained for children of different ages revealed significant differences between the age groups [*F* (11, 343) = 4.58; *p* < 0.001;* η* ^2^ = 0.13]. Post hoc Bonferroni tests indicated that scores of children aged 6 were significantly lower than scores of children aged 11–15 (all *p*s < 0.05). Children aged 11 scored significantly higher (*p* < 0.01) than children aged 5–7. We found no significant main effect of “sex” [*F* (1, 332) = 2.30; *p* = 0.13;* η* ^2^ = 0.01].

As previous reports on normative values in the SST rather present children as one, homogenous group of people below 15 years of age [5–9], the presented results of young children can be used as a guideline for future experiments.

### Relationship between self-assessed sensitivity and identification score

Out of all participants who took part in testing, 722 participants assessed their overall olfactory sensitivity and 710 people assessed their nasal patency. 258 people rated their sense of smell as good or very good, 385 as average, and 63 as bad or very bad. Furthermore, 258 people rated their nasal patency as good or very good, 371 as average, and 81 as bad or very bad.

Generally, the performance in the identification test did not depend on the self-assessed olfactory abilities of the participants [*F* (2, 703) = 2.60, *p* = 0.08; one-way ANOVA]. However, this was due to a nonsignificant effect in the youngest age group, which suggests that only adults could accurately rate their smell. When only the results of people from age groups 2–4 were analyzed (477 participants aged >15), the effect of self-assessed olfactory abilities on identification score was significant [*F* (2, 474) = 6.22, *p* = 0.002]. Post hoc Bonferroni tests pointed toward significant differences between the groups. The results of the group who assessed their sense of smell as bad or very bad (*n* = 57, *M* = 12.61) were significantly lower than results of groups that assessed their smell as good or very good (*n* = 182, *M* = 13.52, *p* = 0.001) and scores of the group of average smellers (*n* = 238, *M* = 13.36 *p* = 0.009). However, the results of “good smellers” and “average smellers” were not significantly different. The participants’ score in identification test was not dependent on their self-assessed nasal patency (*p* = 0.57, one-way ANOVA). This analysis could not be performed for separate age groups because there were no subjects in some subclasses (e.g., people below 16 who assessed their nasal patency as average). Self-ratings of olfactory sensitivity and nasal patency were significantly correlated (*r* = 0.31, *p* < 0.001).

## Discussion

The present investigation revealed the following major results: first, consistent with previously published reports [5, 6], we found that SST identification scores of the youngest and the oldest participant groups were lower than the scores obtained by people aged 20–60. Secondly, we observed an age-related increase in the olfactory abilities of children. Moreover, the self-assessed olfactory abilities were related to SST identification scores, but only in adults, and self-assessed nasal patency was not related to the SST identification score.

In line with previous work [10, 11], performance of men and women in our sample was not significantly different which is in contrast to many publications (for review: [12]). As the currently used odor identification test is designed to be a relatively simple screening test, it might be not sufficient to detect subtle differences between subjects (and thus the sex differences). Also, some studies show that female superiority in terms of olfactory function decreases when men are provided with some help in the retrieval of odor names [13], so it is possible that generally the SST identification subtest might be equally easy or difficult for men and women.

As for the results concerning age-related differences in abilities to identify odors, our data revealed the same pattern of results like previous reports [e.g., 6, 14]. Generally, scores obtained in the oldest and the youngest subjects were decreased in comparison to the participants aged 20–60 years, with scores of the participants aged ≥70 and <10 being the lowest. Numerous articles explain the deterioration of the sense of smell in old people (also termed “presbyosmia”) [15–19]. Lower olfactory abilities might be a result of many factors, including neurodegenerative diseases or cumulative damage to the olfactory epithelium from repeated infections [4, 20]. The reason for worse performance of children in the olfactory tests seems to be less clear and different than in the case of older people—it seems rather unlikely that they result from common causes of disorders in older age, especially when considering that olfactory disorders are relatively uncommon in children [21, 22]. However, the issue of children’s poor olfactory performance has been analyzed in relatively few studies.

Generally, olfaction in children seems to be very good, including sensitivity to body odors [23–26]. Many studies have shown that children can detect, discriminate, and respond to odors and that they can do it starting from the very beginning of their lives. Olfaction might be an important source of information about food, environment, and people [for a review, 27]. Additionally, it is possible to perform olfactory classical conditioning in newborns, which suggests that the sense of smell is already fully functional during later stages of gestation [28]. Still, the children’s sense of smell and/or their verbal abilities seem to develop with age [27, 29]. Therefore, we suggest that the two sources of the age-related differences in olfactory performance of children are their knowledge of odors and their cognitive abilities.

First, performance in odor identification test relies on prior exposure to and familiarity with the target odors [30, 31] and provided response alternatives [33]. Generally, olfactory thresholds of children and young adults are not very different [23, 24]. In the age range of 4–10 years, abilities to name and recognize odors are less developed than those of adults [33], even though children have a substantial odor vocabulary [34]. Hence, the main source of the observed differences between children and adults could be the lack of the odor-specific knowledge in children.

Odor identification appears to be not only an indicator for olfactory function but also for cognitive abilities [15, 35, 36]. The ability to identify olfactory stimuli is significantly correlated with measures of memory, language, and other cognitive abilities [32, 37]; identification involves detection, discrimination, recognition, and retrieval of a name [38, 39]. Therefore, the second source of the age-related differences in olfactory performance in the identification test might be the level of development of cognitive abilities. It has been shown that individual’s cognitive profile exerts a significant influence on higher order olfactory performance [37, 40, 41]. Also, increased olfactory identification was speculated to be a result of, among others, general semantic knowledge and good verbal skills [37, 41, 42], which are lower in younger children [43]. Additional limitations in the cognitive abilities of children which might be important in olfactory testing include difficulties in task comprehension and low concentration abilities [44].

Children almost always perform worse than adults on higher cognitive tasks, but the child’s brain undergoes many changes throughout adolescence [for a review, 45]. In general, the sequence of changes taking place within the brain parallels cognitive development [45–49]. Regions related to primary, motor and sensory systems mature earliest, followed by cortices associated with basic language skills and spatial attention. Higher order association areas, responsible for integration of the primary sensorimotor processes and modulation of basic attention and language processes, seem to mature last [47, 48]. These developmental changes in cortical development have been found to correlate with behavioral performance measures [for a review, 45]. Therefore, lowered (but continuously developing) cognitive abilities of the children might be related to their performance in the identification task. This seems to be reflected in our results, depicting the gradual, age-related increase of the SST identification score in children.

There exist some specific olfactory tests used for children. Some of them—based on identification—resemble the “standard” tools for adults [50, 51], and some are modified a lot, to be more child friendly. For example, the 11-item smell wheel (Sensonics, Inc., Haddon Heights, NJ, USA, [21]) seems to be a game that consists of a rotating cardboard wheel containing microencapsulated ‘‘scratch and sniff’’ odorants and labels and the “Candy Smell Test” (CST, 23 aromatized sorbitol candies; [52]), based on retronasal smelling of aromas combined with a sweet taste is pleasant for children because they may enjoy candies. Still, these tests show the influence of age on the number of recognized odors. There exist also other measures, less dependent on identification and age of the patient, like the MODT (match-to-sample odorant discrimination task) created by Richman and collaborators [53]. However, great variability in the methodology of the existing tests reduces the effectiveness and reliability of their results [54].

The main focus of all the measures designed for children seemed to be brevity and easy application. It would be useful to work on a test that could be included in the standardized and established toolkit, like the SST [6]. There are a few issues that would need to be taken into account when creating such a test. First, children tend to repeat adults’ statements and to respond affirmatively to positively phrased questions [44], thus special consideration has to be given to the phrasing of the questions. Second, although previous results have indicated that pictures may not be very helpful in an odor identification task [5], children might reach higher scores in olfactory tasks when verbal abilities are involved in the test to a lesser degree [e.g., 32, 55], and as they might have limited abilities to read [43], possible answers should be provided as both pictures and words. Also, although in odor identification tests adults and older children typically outperform younger children [6, 56, 57], it is possible to find odors that are familiar to young children—e.g., in the smell wheel [21] bubblegum was well identified by participants of all ages; this suggests that the preselection of odorants for the child-friendly test needs to be particularly careful. Meanwhile, the best solution for proper olfactory examinations conducted by psychophysical methods seems to be the application of child-friendly tools for screening of the olfactory function, and usage of specific age-related norms in well-established methods like SST when longer and more detailed testing is necessary. In summary, the low scores of children might be an artifact, related to their poorer cognitive abilities and insufficient knowledge of odorants applied in the test. Odor identification tests require special adaptation before they can be administered in various populations [10, 14, 58–60]; however, most existing studies rather have not analyzed the problems associated with application of this test in children.

Interestingly, our participants could only assess their own olfaction to the extent that the group who assessed their sense of smell as bad or very bad scored lower in the test than the groups that assessed their smell as average, good, or very good. However, the scores of “average” and “good” groups were not significantly different. Our results and previous reports [61, 62] suggest that, especially in the case of people who think that their olfaction is good or very good, self-assessments of olfactory function are rather unreliable. In a study of Landis and collaborators [61] the self-assessments of olfactory abilities rather reflected changes of nasal airway patency than measurable olfactory function (but the ratings were accurate when they were performed after olfactory testing). The children in our study were not able to accurately assess their own olfactory sensitivity in any case. This might suggest that the question or the applied Likert scale were so difficult that they were not able to fully understand and complete the task. In future studies regarding self-assessed olfactory abilities in children it would be helpful to add, e.g., graphical emotion clipart to better illustrate the presented choice options.

The present study has some limitations. First, we tested only one olfactory modality and—due to time constraints—did not perform extended testing of various olfactory functions [63, 64]. Second, we only analyzed a very rough medical history and we did not perform any cognitive tests with the subjects. However, the participants did not report major diseases that would affect the olfactory system (e.g., acute or chronic nasal affections; diabetes mellitus, or liver disease) and as we collected the data during scientific fairs, selection of participants was rather biased toward well-informed, interested people who were very likely not to be demented.

In summary, our study showed that—consistent with previously published reports—identification scores of the youngest and the oldest participants were lower than the scores obtained by people aged 20–60. Furthermore, olfactory abilities of children were increasing with age and it seems that the sources of the age-related differences in identification test results relate to knowledge of odors and cognitive abilities. Additionally, we showed that the self-assessed olfactory abilities were related to actual performance in the smell test, but only in adults, and self-assessed nasal patency was not related to the “Sniffin’ Sticks” identification score.
